# Antimony Immobilization in Primary-Explosives-Contaminated Soils by Fe–Al-Based Amendments

**DOI:** 10.3390/ijerph19041979

**Published:** 2022-02-10

**Authors:** Ningning Wang, Yucong Jiang, Tianxiang Xia, Feng Xu, Chengjun Zhang, Dan Zhang, Zhiyuan Wu

**Affiliations:** 1 Beijing Key Laboratory for Risk Modeling and Remediation of Contaminated Sites, National Engineering Research Center of Urban Environmental Pollution Control, Beijing Municipal Research Institute of Eco-Environmental Protection, Beijing 100037, China; wangningning@cee.cn (N.W.); zhangdan@cee.cn (D.Z.); wuzhiyuan@cee.cn (Z.W.); 2 Beijing Institute of Mineral Resources and Geology, Beijing 101500, China; jiangyu_cong@163.com; 3 Technical Centre for Soil, Agriculture and Rural Ecology and Environment, Ministry of Ecology and Environment, Beijing 100012, China; xufeng@tcare-mee.cn; 4 State Key Laboratory of Water Environment Simulation, School of Environment, Beijing Normal University, Beijing 100875, China; zhangcj@mail.bnu.edu.cn

**Keywords:** primary explosives site, heavy metal contamination, antimony, co-occurring metal, Fe–Al-based amendment, immobilization

## Abstract

Soils at primary explosives sites have been contaminated by high concentrations of antimony (Sb) and co-occurring heavy metals (Cu and Zn), and are largely overlooked and neglected. In this study, we investigated Sb concentrations and species and studied the effect of combined Fe- and Fe–Al-based sorbent application on the mobility of Sb and co-occurring metals. The content of Sb in soil samples varied from 26.7 to 4255.0 mg/kg. In batch experiments, FeSO_4_ showed ideal Sb sorption (up to 97% sorption with 10% FeSO_4_·7H_2_O), whereas the sorptions of 10% Fe^0^ and 10% goethite were 72% and 41%, respectively. However, Fe-based sorbents enhanced the mobility of co-occurring Cu and Zn to varying levels, especially FeSO_4_·7H_2_O. Al(OH)_3_ was required to prevent Cu and Zn mobilization. In this study, 5% FeSO_4_·7H_2_O and 4% Al(OH)_3_ mixed with soil was the optimal combination to solve this problem, with Sb, Zn, and Cu stabilizations of 94.6%, 74.2%, and 82.2%, respectively. Column tests spiked with 5% FeSO_4_·7H_2_O, and 4% Al(OH)_3_ showed significant Sb (85.85%), Zn (83.9%), and Cu (94.8%) retention. The pH-regulated results indicated that acid conditioning improved Sb retention under alkaline conditions. However, no significant difference was found between the acidification sets and those without pH regulation. The experimental results showed that 5% FeSO_4_·7H_2_O + 4% Al(OH)_3_ without pH regulation was effective for the stabilization of Sb and co-occurring metals in primary explosive soils.

## 1. Introduction

Antimony (Sb) is an element of growing environmental concern because of the widespread use and uncontrolled release of Sb compounds into the environment [1,2,3,4]. In addition, antimony has a carcinogenic potential [5] and Sb(III) has been shown to potentially cause lung cancer in female rats [6]. Background concentrations of Sb in soil tend to be lower than 1 mg/kg [3]. However, the major smelting and general use of Sb has led to severe site contamination, which poses a significant risk to the local environment [7,8,9] and humans [10]. An increasing number of studies have shown that antimony pollution is a global issue [11,12] because of its toxicity to humans and its role in causing liver, skin, and respiratory and cardiovascular diseases [10]. Soil is an important medium for Sb concentration and migration. In recent years, increased attention has been given to Sb-contaminated soil in mining areas and shooting ranges [10,13,14,15,16]. However, few studies have investigated Sb-contaminated soils by primary explosives because of confidentiality and sensitivity. A significant input of Sb into the environment occurs through the production and use of primary explosives because Sb was historically used as a combustible agent for classical primary explosives, which contain 33.4% Sb_2_S_3_ [17]. The weathering and corrosion of combustion residue lead to the mobilization of metalloid Sb in anionic form [18]. Primary explosives sites, which are often characterized by critical concentrations of co-occurring copper (Cu) and zinc (Zn) [19,20,21], can be of particular environmental concern because they represent hazardous multi-element contamination sources for sites zones. The leachate of Sb and co-occurring metals from primary explosive production and use areas poses a serious long-term threat to the environment and human health. Thus, the immobilization or a reduction in the mobility and bioavailability of Sb and co-occurring metals in primary explosives sites is critical.

Sb enters the soil environment primarily as Sb_2_S_3_ (stibnite) and Sb_2_O_3_ (senarmontite, valentinite) [22]. Chemical/physical weathering and naturally occurring oxidation and microbial processes are often responsible for converting primary Sb mineral phases, predominantly sulfides and oxides, to secondary Sb minerals, which are soluble in soil pore water and more mobile in the environment [23]. Most of the antimony is confined to the top 30 cm layer of the soil and is bound tightly to soil-derived humic acid molar mass fractions that are extracted from the top 10 cm layer [12]. The formation of stable secondary Sb minerals by precipitation and adsorption on metal oxyhydroxides (for example Fe-based sorbents [24,25]) are the most prominent naturally occurring processes that can control the mobility and transformation of Sb species in soil systems [26]. In the natural environment, the mobility, bioavailability, and toxicity of Sb are primarily dependent on its chemical speciation [3,27]. Antimony exists in a variety of oxidation states (−III, 0, III, V), with oxidation states III and V being predominant in aqueous environments across a wide pH range (4–10) [28]. In the natural environment, Sb(III) occurs primarily as Sb(OH)_3_ under anaerobic conditions between pH 2 and 10 [3,27], whereas Sb(V) is a predominant species that exists as Sb(OH)_6_^-^ in aerated environments [29] and has a high affinity to amorphous and crystalline Fe-(hydr)oxides, with which it can form stable bidentate inner-sphere complexes [30]. These interactions are favored by goethite in the pH range of 7.5–9.0, by hematite at pH 8.5, by ferrihydrite in the pH range of 7.0–7.9, and by akaganeite in the pH range of 9.5–10 [31]. However, Sb with different valence states has different properties; for instance, Sb(III) can adsorb strongly on goethite over a wide pH range from 3 to 12, whereas the maximum adsorption of Sb(V) occurs only below pH 7 [32]. These Fe-based metals adsorb Sb strongly and act as oxidants to transform Sb(III) to Sb(V) [33,34]. Laboratory-scale testing indicated that Fe_2_(SO_4_)_3_ may be applicable to Sb immobilization in soils [15]. The sorption effect is based on the reaction of Sb(V) with the surface hydroxyl group of Fe-based materials. However, the mobility of co-occurring Cu and Zn was enhanced after Fe-based sorbent addition [15]. In the pH range of 5–9, co-occurring metals behave differently, and are commonly present in soil solutions as cations at acidic and circumneutral pH or as soluble soil organic matter (SOM)-metal(II) complexes at higher pH [31]. At neutral and alkaline pH, substantial amounts of heavy metals are immobilized as Me-hydroxides, Me-caralbonates or Me-hydroxycarbonates (Me indicates heavy metals). Soluble heavy metals show a limited affinity for hydroxyl groups because of their cationic nature, but they interact more strongly with negatively charged components [31]. Thus, the different speciation, mobility, and bioavailability between Sb and co-occurring metals make the identification of suitable amendments challenging. Aluminum (hydr)oxides show important sorption properties for Pb, Cd, and Zn [35]. Aluminum (hydr)oxides can be protonated, which makes their surface positively charged and generates electrostatic interactive forces with negatively charged Sb(V) [36]. Only a few amendments, which are based mostly on Fe- and Al-containing materials, have been tested with variable success as Sb-immobilizing agents. Limited studies have examined the mobility of Sb and co-occurring metals in soil and the selection of ideal sorbents for their immobilization.

The focus of this work was on soil that was contaminated with Sb in primary explosives production sites with the main goals being: first, to investigate the mobility and speciation of Sb in primary explosive sites; second, to evaluate the effect of the combined application of Fe–Al mixed amendments for primary explosive sites using batch and column tests; and third, investigate the pH effect on Sb immobilization.

## 2. Materials and Methods

### 2.1. Sb-Contaminated Soils and Adsorbents

Sb-contaminated soils were collected from primary explosives production workshops from a primary explosives site in Heilongjiang, China. The specific coordinates have not been provided because of the confidentiality and sensitivity of this military enterprise. This site produced primary explosives for more than 60 years and remains operational. Based on previous environmental site investigation by soil boreholes, it was found that Sb was enriched mostly on the surface soil layer, and thus, nine soil samples (S1–S9) were collected at the surface (0–20 cm depth). Samples were air-dried at room temperature, crushed with a wooden roller and sieved through a 150-μm mesh, which is thought to better estimate human exposure than bulk soil [37]. The samples were mixed and prepared for soil analysis.

The soil sample with the highest concentration of Sb was used for soil immobilization studies. Four adsorbents were used: ferrous sulfate (FeSO_4_·7H_2_O, powder); goethite (HFeO_2_, powder); Fe^0^ (powder); and aluminum hydroxide (Al(OH)_3_, powder).

### 2.2. Soil Analysis

Soil physicochemical properties were measured on triplicate samples with three blanks. Soil pH was measured in a 1:5 (*w*/*v*) soil/deionized soil suspension by using a pH meter (PHSJ-4A, INESA Scientific Instrument Co., Ltd., Shanghai, China) after 1 h of equilibration according to ISO10390:2005. After microwave-assisted digestion with HCl + HNO_3_ + HClO_4_ (3:1:1) at 190 °C for 15 min, the mixture was cooled, filtered (<0.45 μm), and diluted with ultrapure deionized water. Total Sb (Sb_tot_) concentrations were determined by hydride generation atomic fluorescence spectrometry (HG-AFS) (AFS 9700, Titan Instrument Co. Ltd., Beijing, China) [2]. The total contents of Fe, Mn, Zn, and Cu were determined by using inductively coupled plasma atomic emission spectrometry (ICP-AES) (NexION300x, PerkinElmer, Waltham, MA, USA) [38]. The modified European Community Bureau of Reference (BCR) sequential extraction method [39] was used as a sequential extraction process for Sb. Sb speciation in soil is divided into a soluble/exchangeable fraction (F1), reducible fraction (F2), oxidizable fraction (F3), and residual fraction (F4) based on the BCR technique [40]. The processes were as follows—step 1: 0.5 g soil + 20 mL of 0.11 mol/L HAc was agitated continuously at ambient temperature for 16 h, and supernatant was used to determine the acid-soluble fraction; step 2: the soil in step 1 + 20 mL of 0.5 mol/L NH_2_OH·HCl was agitated at ambient temperature for 16 h, and the supernatant was used to determine the reducible fraction; step 3: the soil in step 2 + 5 mL of 8 mol/L H_2_O_2_ was agitated continuously at ambient temperature for 1 h, placed in a water bath at 85 °C for 1 h and treated with 25 mL of 1 mol/L NH_4_Oac for 16 h at ambient temperature, with supernatant being used to determine the oxidizable fraction; step 4: the residual soil was digested with aqua regia to obtain a residue fraction.

### 2.3. Immobilization Experiment

#### 2.3.1. Batch Experiments

Batch experiments and column leachate experiments were performed on the soils that were most polluted with Sb (S9). Experiments were performed in a 50 mL centrifuge tube with four grams of untreated soil or soil amendment mix (0%, 2%, 5%, and 10% iron-based adsorbent; 0%, 2%, and 4% aluminum-based adsorbent) and the corresponding volume of deionized water was treated with a Milli-Q water purification device (Millipore Corp, Billerica, MA, USA) with a liquid-to-solid ratio of 10 (L/S). Centrifuge tubes were shaken at 100 rpm/min for 10 days at room temperature (25 ± 1 °C) in an incubator shaker. Based on the earlier sorption studies of Sb to Fe-based materials, 10 days reaction time was sufficient to reach equilibrium [15,41]. All experiments were performed in triplicate. After reaction, the samples were centrifuged, and the supernatant was filtered with a 0.45 μm filter membrane (PES, ReLAB). The filtrate was used for Sb, Cu, and Zn analysis.

#### 2.3.2. Column Leachate Experiments

**Figure 1:**
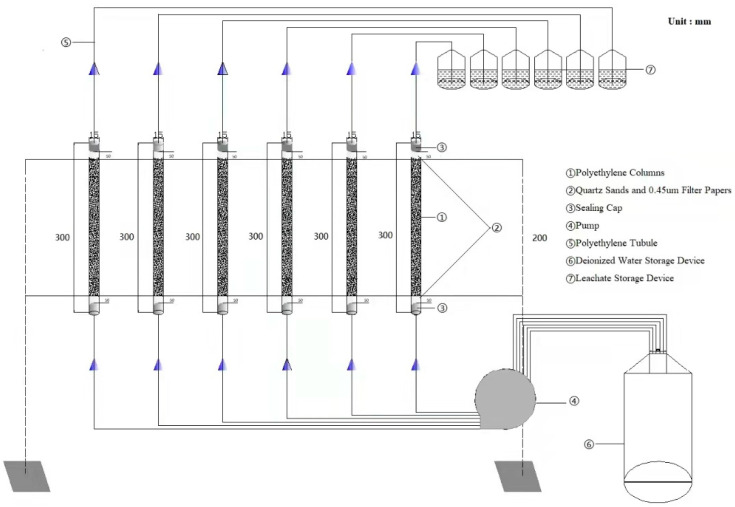
Structure of column experiments.

Column leachate experiments were carried out in triplicate with untreated soil and in quintuplicate with 5% FeSO_4_ and 4% Al(OH)_3_ stabilized soil without or with pH regulators (i) 4% sodium bisulfate and (ii) 4% sodium carbonate. Column leachate experiments, as shown in Figure 1, were carried out in polyethylene columns (length 300 mm × internal diameter 22 mm) packed with 160 g thoroughly mixed soil material of untreated soil (Group A), stabilized soil + 4% NaHSO_4_ (Group B), stabilized soil (Group C), and stabilized soil + 4% Na_2_CO_3_ (Group D).

Double filter papers (0.45 um) and 50 mm long quartz sands were installed at the top and bottom of the column for particle retention. Deionized water was added to the columns in an upward flow direction at approximately 0.38 mL/min by a peristaltic pump over an 18-day period. The calculated pore volume (PV) in the soil column was 0.043 L, based on an assumed soil porosity of 0.4 (total 550−600 PV) [15]. During the column experiment, samples were collected regularly and filtered for immediate analysis.

## 3. Results and Discussions

### 3.1. Soil Characteristics and Risk

The main soil physicochemical properties are summarized in Table 1. All nine soil samples were calcareous, with pH values varying from 7.69 to 8.37, which is consistent with the local soil pH range [42]. The concentrations of Fe and Mn in the soil were 2 × 10^4^–3.22 × 10^4^ mg/kg and 396–719 mg/kg, respectively. The total concentration of Sb, Cu, and Zn in the studied area varied from 26.73 to 4255, 24.29 to 312.3, and 67.62 to 1330 mg/kg, respectively. Antimony was the main contaminant in this primary explosives site and was 0.34–211.8 times higher than the Chinese screening value of the first land use category (20 mg/kg), based on the document of “Soil Environmental Quality Risk Control Standard for Soil Contamination of Development Land”, which was issued by the Ministry of Ecology and Environment of China. The highest concentration of Sb in soil sample S9 (4255 mg/kg) was 211.8 times and 105.4 times higher than the corresponding risk screening value (20 mg/kg) and control value (40 mg/kg), respectively.

**Figure 2:**
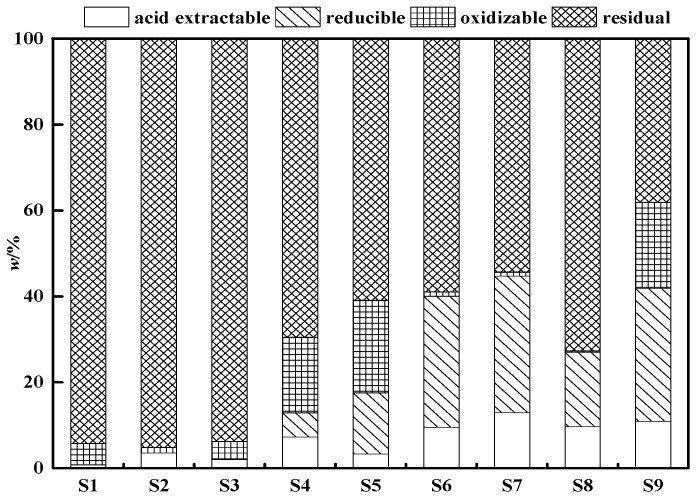
Sequential extraction of Sb from primary-explosives-contaminated soils.

The BCR method is used extensively as a sequential extraction process for heavy metal [38]; thus, BCR sequential analysis was used to measure the main contaminant (Sb). The fraction of Sb in the primary-explosives-contaminated soil in Figure 2 followed the order (average values): F4 fraction (38.05–94.22%) > F2 fraction (0.01–31.80%) > F3 fraction (0.32–21.55%) > F1 fraction (0.76–12.92%). The F4 fraction of the heavy metals is considered to be associated with stable minerals with the lowest mobility [40]; thus, Sb in S2 soil sample was the most stable. As a result, S2 may pose least risk. The F4 fraction of Sb was slightly different in S1, S2, and S3, and decreased significantly in the other soils, especially in S9 (only 38.05%). The Sb of the F1 and F2 fractions can be combined as directly available fractions [43] with a direct toxicity [44] because the Sb in these two fractions is highly mobile when environmental conditions (such as pH and Eh) change. The F3 fraction of Sb is easily mobilized and transformed into the F1 and F2 fractions, and potential eco-toxicity should not be ignored [44]. The total concentrations of F1, F2, and F3 fractions are a bioavailable fraction because of their direct and potential eco-toxicity [40]. Figure 2 shows that Sb had the highest proportion of F1 + F2 + F3 phase (61.95%) in S9, which indicates that S9 had a high migration potential and the greatest biological impact [36]. The amount of exchangeable Sb is negatively correlated with the concentration of Fe (r = −0.867, *p* < 0.01) for all soil samples.

### 3.2. Batch Experiments

#### 3.2.1. Changes in pH and Sb Fractions

**Figure 3:**
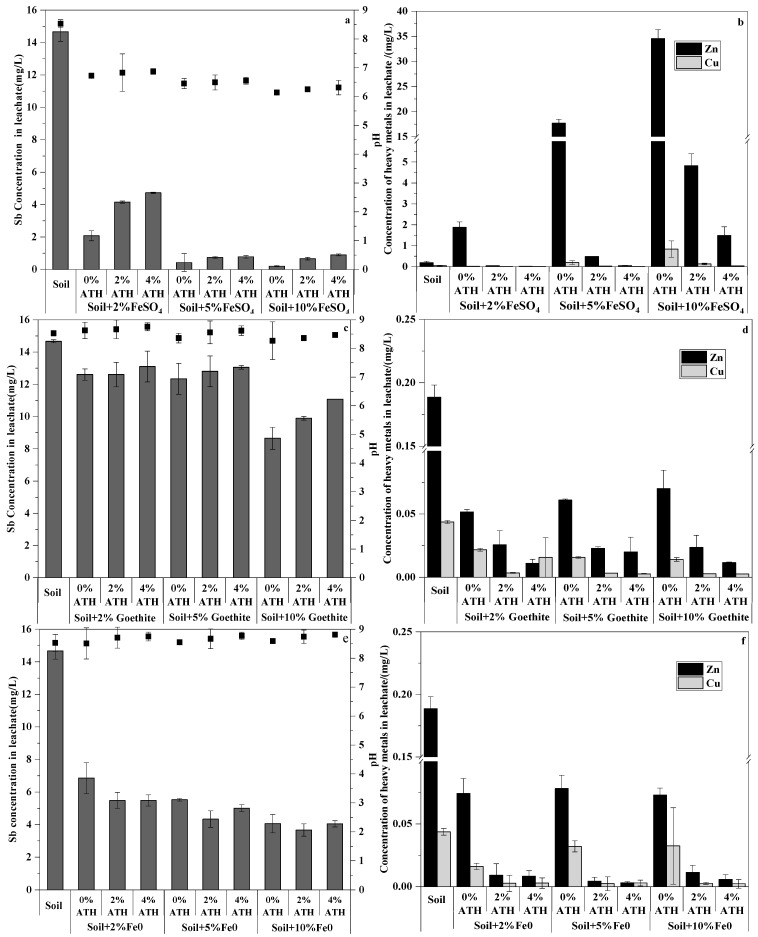
pH and Sb and co-occurring heavy metals concentration in water extracts ((**a**,**b**)—FeSO_4_ + ATH; (**c**,**d**)—Goethite + ATH; (**e**,**f**)—Fe^0^ + ATH).

We evaluated the application of ferrous sulfate (FeSO_4_·7H_2_O), goethite (HFeO_2_), and Fe^0^ at three different percentages (2%, 5%, and 10%), as potential amendments for the remediation of Sb and soil contaminated with co-occurring metals. To obtain stabilizing results, Al(OH)_3_ was applied (0%, 2%, and 4%) as combined amendments with Fe-based amendments. As shown in Figure 3, after the equilibration period of 10 d, FeSO_4_·7H_2_O addition significantly decreased the water extract pH (21%, 24%, and 28% decreased at 2%, 5%, and 10% amendment percentages, respectively) compared with the control, whereas HFeO_2_ and Fe^0^ had little effect on the water extract pH. This behavior is a result of the hydrolysis reaction of FeSO_4_ (Fe^2+^ + 2H_2_O ⇌ Fe(OH)_2_ +2H^+^) in the pore water of soil [45,46]. The application of Al(OH)_3_ increased the water extract pH in a small range (less than one unit) regardless of the combined application with FeSO_4_·7H_2_O, HFeO_2_, or Fe^0^. This slight increase in pH is attributed mainly to the weak alkalinity of Al(OH)_3_ and partial dissolution of amendments and adsorption/precipitation [47].

**Figure 4:**
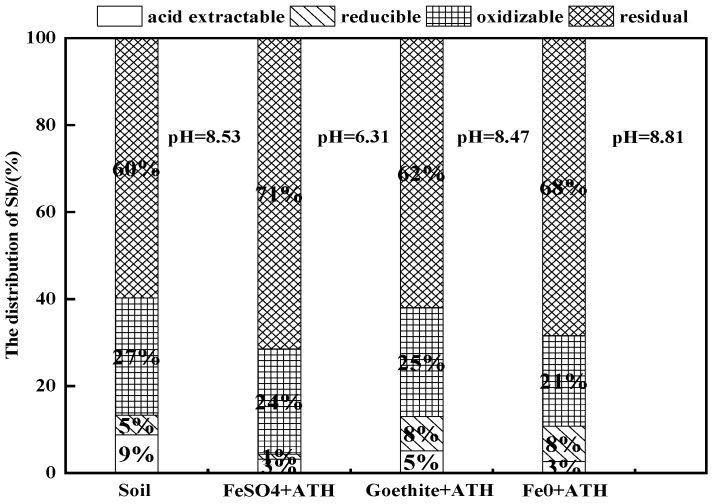
Sequential extraction of Sb in amended soil with Fe and Al mixed adsorbent.

Sequential BCR extraction procedures were used for 5%Fe + 4%Al-based modified soils (with the best modified result) and are presented in Figure 4. The Sb proportions of F1, F2, F3, and F4 fractions in the control soil were 8.7%, 4.5%, 27.0%, and 59.7%, respectively. The F1 fraction [40] decreased significantly in modified soils compared with the control. Approximately 66.67%, 44.44%, and 66.67% reductions (vs. control) were observed for FeSO_4_ + Al(OH)_3_-, goethite + Al(OH)_3_-, and Fe^0^ + Al(OH)_3_-modified soil, respectively. This result is important because this fraction of Sb has the most biological impact with a direct toxicity [44]. With F1, F2 fractions are considered the most available to soil biota and the most easily leached to groundwater [43]. However, only FeSO_4_ + Al(OH)_3_ addition reduced the F2 fraction from 5% to 1%, despite a decrease in soil pH. The decrease in soil pH was likely caused by an increase in exchange sites (Fe/Al oxides and oxyhydroxides), which has a great affinity for Sb [48]. Fe–Al-based amendment additions induced a shift of F1, F2, and F3 fractions towards to F4 fraction, which was more strongly retained by the Fe and Al (hydr)oxides [29,49] by adsorption. The concentration decrease in the F1, F2, and F3 fractions resulted in a subsequent low bioavailability and eco-toxicity [40]. However, this result was not clear for the goethite- and Al(OH)_3_-modified soil with only a 2% bioavailable fraction shift to a stable residual fraction. FeSO_4_ + Al(OH)_3_ addition caused a significant increase in F4 (71%) compared with goethite + Al(OH)_3_ and Fe^0^ + Al(OH)_3_ groups, which could explain its high sorption capacity of Sb [50]. Overall, the BCR extract results showed that the combined addition of 5%FeSO_4_ and 4%Al(OH)_3_ induced a significant redistribution of Sb with a reduction in its more labile and bioavailable fraction and an increase in the residual fraction.

#### 3.2.2. Effects of Fe- and Al-Based Sorbents on Sb

The results of the batch experiments (Figure 3) showed that the leachate concentration of Sb in the control groups exceeded 14 mg/L, which is higher than the fifth category water limit (0.01 mg/L) of the “Standard for groundwater quality” issued by the General Administration of Quality Supervision, Inspection and Quarantine of the People’s Republic of China (AQSIQ, 2017). This result proved a high liquidity of antimony in primary-explosives-contaminated soils and occurs mainly because of the high percentage of soluble fraction (11.03%) and reducible fraction (30.12%) of Sb in S9 (Figure 2). Thus, it is necessary to immobilize or reduce the mobility and bioavailability of Sb in explosives-contaminated soils to reduce plant and human bioavailability and Sb leachate in groundwater [51].

The amendment additions did not change the total Sb concentrations. However, Fe-based amendments prevented Sb mobility and reduced Sb bioavailability by a strong preference of Sb binding to Fe hydroxides [52]. FeSO_4_·7H_2_O possessed the strongest sorption properties for Sb with 85.64%, 97.21%, and 98.50% sorptions with 2%, 5%, and 10% additions, respectively (Figure 3a). The sorption property was enhanced by the addition of various concentrations of FeSO_4_·7H_2_O. The sorption increased gradually with an increase in FeSO_4_·7H_2_O concentration from 0% to 10% and was moderate with FeSO_4_·7H_2_O concentrations from 5% to 10%. The amendments of Fe^0^ and HFeO_2_ showed a lower Sb sorption compared with FeSO_4_·7H_2_O. The sorption increased gradually with an increase in Fe^0^ or HFeO_2_ concentration from 0% to 10%; 10% Fe^0^ was retained up to 72.34% Sb (Figure 3c), and the HFeO_2_ only retained up to 41.05% Sb (Figure 3e). In the severely Sb-contaminated primary explosives site, FeSO_4_·7H_2_O was more effective for Sb immobilization than Fe^0^ or HFeO_2_. The results are consistent with the immobilization of Fe-based amendments for Sb in shooting range soil [15]. On the basis of the thermodynamics principle, Sb(V) should be the main form in oxic environments [15,53]. In addition, widely distributed dissolved iron in the environment impacts rapid Sb(III) oxidation [54]. Ferric ion and iron oxyhydroxides in the environment are strong Sb adsorbents [30]. Sb(III) can be oxidized simultaneously into Sb(V) once adsorbed on the iron oxyhydroxide surface [54]. The immobilization mechanism of iron oxide for Sb(V) was summarized as direct precipitation, co-precipitation, and adsorption [55,56]. The direct precipitation mechanism could lead to secondary Fe–Sb mineral tripuhyite (FeSbO_4_) formation, which is an important and ultimate sink for Sb in an environment with a low solubility (log Kso = −13.41) [57]. Sb(V) adsorption was a predominant mechanism, and co-precipitation was important in FeSO_4_·7H_2_O-modified soil [58]. FeOOH adsorption and the hydrolysis product of Fe(II) and Fe(III), rather than co-precipitation, was predominant in the coagulation mechanism [30]. The Fe(II) in the solution was oxidized rapidly to Fe(III), which improved the antimony removal efficiency [46]. Fe(II) oxidation and ferric-hydroxide formation may lead to an increase in the adsorption between ferric flocs and Sb [59,60]. FeSO_4_·7H_2_O addition can reduce the soil solution pH, and at pH < 7, iron oxide showed a strong affinity to Sb. When pH < 3, this level [H^+^] could inhibit the extent of Fe(II) and Fe(III) hydrolysis, which limited the Sb(V) immobilization efficiency. For pH 3–6, the isoelectric point of FeSO_4_-produced iron flocs was 7.5. Iron flocs with a positive charge had a better capture of negatively charged Sb(OH)_6_^-^ at a weak acid condition and for pH 5 to 6 [58]. As a result, FeSO_4_·7H_2_O amendment could promote Sb(V) immobilization by reducing the soil solution pH. The maximum Sb(V) adsorption on HFeO_2_ existed below pH 7 [32] and was considered pH-dependent [61]. In this study, the soil extract solution pH exceeded 7; thus, HFeO_2_ immobilized Sb in these primary-explosives-contaminated soils to a certain extent. When in contact with oxygenated water, Fe^0^ converts to activated Fe^0^ that contains ferrihydrite and goethite, which are distributed on the surface [4]. The large surface coverage makes activated Fe^0^ to be recognized as a suitable adsorbent for Sb adsorption and immobilization [62]. However, the immobilization efficiency of Fe^0^ for Sb was lower than that for FeSO_4_·7H_2_O. According to batch experiments of Fe-based amendments, FeSO_4_·7H_2_O was most appropriate for high concentrations of Sb-contaminated soil.

Al(OH)_3_ addition slightly increased Sb mobility regardless of FeSO_4_-, goethite-, or Fe^0^-modified soils by increasing the soil pH [58]. The sorption of Sb in FeSO_4_- and Al(OH)_3_-modified soil decreased slightly from 85.82% to 67.77% in 2% FeSO_4_, from 97.09% to 94.69% in 5% FeSO_4_, and from 98.60% to 93.88% in 10% FeSO_4_ with Al(OH)_3_ addition from 0% to 4%. Approximately 2.47–21.03% reductions were observed with Al(OH)_3_ addition. Sb sorption in goethite- and Al(OH)_3_-modified soil decreased from 14.09% to 10.68% in 2% goethite, from 15.91% to 11.02% in 5% goethite, and from 41.04% to 24.55% in 10% goethite with an Al(OH)_3_ addition from 0% to 4%. Approximately 24.19–40.20% reductions were observed. However, a change in sorption was not clear for Fe^0^- and Al(OH)_3_-modified soil, even with substantial amounts of Al(OH)_3_ [48]. In the Fe–Al mixed-addition cases, a reduction in sorption may have resulted from the change in pH [63]. Al(OH)_3_ shows a lower adsorption efficiency than iron oxides, especially in neutral and alkaline environments [64]. However, FeSO_4_- and Al(OH)_3_-modified soil still showed a more efficient sorption, which is attributed to the ion-exchange ability of Fe–Al double hydroxides and SbO_3_^−^ adsorption on the FeO(OH) surface [65].

#### 3.2.3. Effect of Fe- and Al-Based Sorbents on Zn and Cu

FeSO_4_·7H_2_O amendment addition to the soil resulted in a high concentration decrease in Sb; however, higher concentrations of Zn and Cu were detected in the soil extract solution, especially for Zn (Figure 3). Zn release increased gradually with an increase in FeSO_4_·7H_2_O concentration from 0% to 10%. After FeSO_4_·7H_2_O addition, the highest leachate concentration of Zn was 34.57 mg/L, which was 181.94 times the original soil leachate concentration (0.19 mg/L). Similar promotion leachate effects of FeSO_4_·7H_2_O were found for Cu. The leachate concentration of Cu was 0.84 mg/L with 10% FeSO_4_·7H_2_O addition, which was 21 times the leachate concentration compared with the original soil (0.04 mg/L). FeSO_4_·7H_2_O addition decreased the water extract pH by the hydrolysis of FeSO_4_ [45,46]. The acid environment promoted Zn and Cu release, which resulted in a high leachate concentration of Zn and Cu [66,67]. In contrast to FeSO_4_, goethite and Fe^0^ application significantly reduced the concentration of Zn and Cu. No further reduction was caused by goethite and Fe^0^ addition. As shown in Figure 3, the maximum adsorption efficiencies of Zn and Cu were 72.60% and 68.05%, respectively, in goethite-treated soil. The maximum adsorption efficiencies of Zn and Cu were 61.29% and 63.31%, respectively, in Fe^0^-modified soil. Zn and Cu fixation by goethite could be attributed mainly to metal diffusion into the structural lattice of goethite by the following reactions: ≡Fe-OH + Me^2+^ + H2O ⇌ ≡Fe-O-MeOH_2_^+^; ≡Fe-O-MeOH_2_^+^ + Me^2+^ + 2H2O ⇌ Fe-O-MeOH_2_^+^ + Me(OH)_2_(s) + 2H^+^ [68]. With Al(OH)_3_ addition, the Zn and Cu concentration in the leachate decreased significantly, especially in FeSO_4_·7H_2_O-treated soil. Al(OH)_3_ (4%) decreased the extractable Zn by 99.72%, 83.47%, and 96.26% in FeSO_4_·7H_2_O, goethite, and Fe^0^-modified soil, respectively (Figure 3), whereas 4% Al(OH)_3_ addition decreased the extractable Cu by 96.11%, 83.61%, and 93.21% in FeSO_4_·7H_2_O, goethite, and Fe^0^-modified soil, respectively. The reduction in extractable Zn and Cu in Fe–Al-based modified soil may indicate that Al(OH)_3_ was important in Zn and Cu immobilization. These results are similar to those reported previously [36] and are somewhat expected in Fe–Al-based modified soil, where a substantial amount of the Sb and co-occurring metals are likely to form stable surface complexes or precipitates with iron and aluminum hydroxide [36]. In soil that is amended with 5% ferrous sulfate and 4% aluminum hydroxide, the leachate concentrations of Sb, Zn, and Cu decreased significantly, and the maximum stabilization efficiencies were 94.69%, 74.17%, and 82.15%, respectively. Goethite (10%) and aluminum hydroxide (2%, pH = 8.36) addition yielded a 32.60% stabilization efficiency of Sb and an decreases of 87.45% and 93.73% in Zn and Cu, respectively. The mixture of 10% Fe^0^ and 2% aluminum hydroxide (pH = 8.74) stabilized Sb, Zn, and Cu in soil with efficiencies of 75.00%, 94.01%, and 94.14%, respectively. For high Sb contamination and light pollution with Zn and Cu, 5% FeSO_4_·7H_2_O and 4% Al(OH)_3_ addition was shown to be the most suitable amendment course.

### 3.3. Column Experiments

#### 3.3.1. Column Experiments for Sb Immobilization

**Figure 5:**
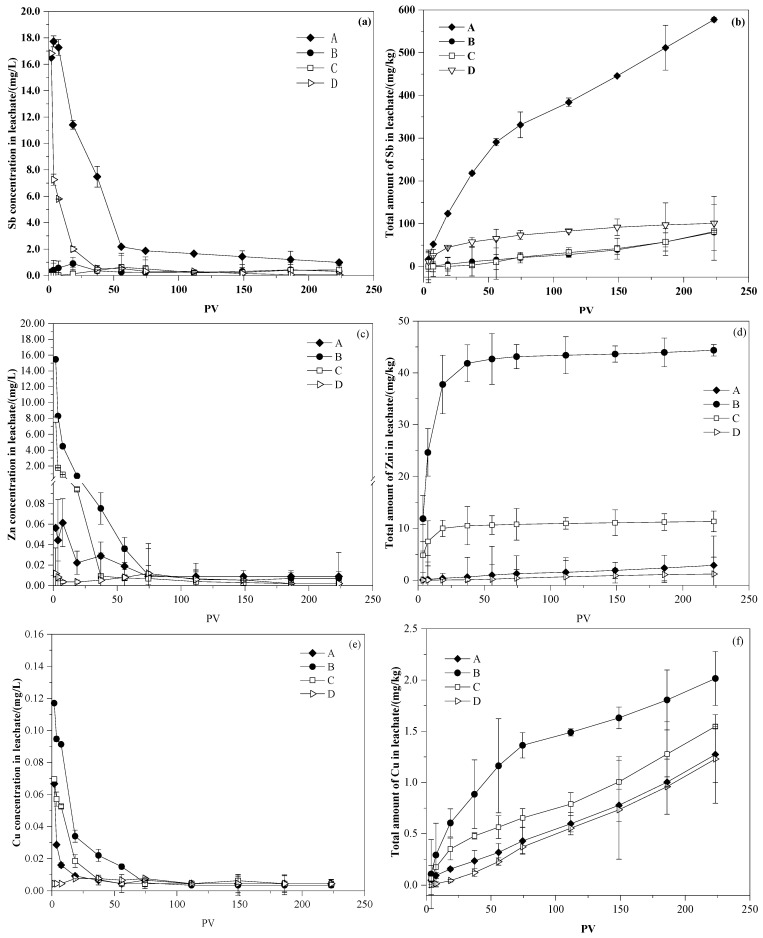
Changes in leachate concentration and accumulation of heavy metals in column experiment: (◆) untreated soil, (●) Fe–Al-stabilized soil with acidification, (□) Fe–Al-stabilized soil, (▷) Fe–Al-stabilized soil with alkalization ((**a**,**c**,**e**)—Sb, Zn, Cu concentration in leachate respectively; (**b**,**d**, **f**)—total leaching amount of Sb, Zn, Cu/ weight of soil).

In column experiments, the initial Sb concentration in leachate of untreated soil was 16.5 mg/L, which exceeds the GB/T 14848—2017 (Standard for Groundwater Quality) type-V leachate limit (0.01 mg/L) (AQSIQ, 2017) (Figure 5a). When the pore volume reached 3.7, the concentration of Sb in leachate increased substantially to a peak of 17.73 mg/L. Similar leachate results were found in shooting range soil [15]. Okkenhaug et al. [15] assumed that this initial increase in Sb occurred because of the depletion of H^+^ in the soil during leachate, which induced a decrease in the positive surface charge of minerals such as iron oxyhydroxides. Consequently, the sorption capacity of negatively charged anions decreased. In this study, no significant pH increase was recorded. Sb mobilization may have increased in untreated soil through the depletion of organic and inorganic components and through the partial dissolution of reducible and oxidizable fractions [1,69]. After an initial increase, the Sb concentration decreased exponentially up to a PV of 74.4. At this stage, the pH decreased rapidly to 7.03. Subsequently, the pH stabilized at ~7. As a result, the Sb concentration stabilized at 0.99 mg/L. The trend in Sb leachate was consistent with the pH trend, which meant that Sb dissolution was affected by soil pH [70]. Hockmann et al. [71] found a similar effect for Sb in large-range soil under large seepage conditions. The initial Sb concentration of leachate in a 5% FeSO_4_·7H_2_O- and 4% Al(OH)_3_-modified column was 0.024 mg/L, which was 675 times lower than that of untreated soil, and the stabilization efficiency was 99.86%. The pH was adjusted to 3–10 by the addition of pH regulators, NaHSO_4_ or Na_2_CO_3_. The leachate concentration of Sb in the stabilized soil is shown in Figure 5a. The leachate concentrations of Sb in descending order was as follows: Group A, Group D, Group B, Group C. Group D showed a high initial Sb concentration (16.82 mg/L) compared with Group B (0.29 mg/L) and Group C (0.02 mg/L) as a result of the rapid increase in pH that was induced by large amounts of OH^−^, which decreased the positive surface charge of iron and aluminum oxyhydroxide. Consequently, Group B decreased the retention capacity of negatively charged anions [67,72]. The leachate concentration of Sb in Group B decreased to levels similar to Group C with an increase in PV and a decrease in pH.

The calculation results of the cumulative leachate amount of Sb in the dynamic process of each group are shown in Figure 5b. When the PV reached 223.3, the total amounts of Sb that was leached in Groups A, B, C, and D were 577.61, 79.75, 81.71, and 101.01 mg/kg, respectively. Soils that were amended with FeSO_4_·7H_2_O mixed with Al(OH)_3_ showed a good Sb stabilization effect [36], with stabilization efficiencies of 86.19%, 85.85%, and 82.51% in Groups B, C, and D, respectively. Group B decreased the soil pH significantly, but did not increase the retention effect of Sb. The pH of Group C was consistent with untreated soil when the PV reached 37.2. The 5% FeSO_4_·7H_2_O and 4% Al(OH)_3_ mixed addition showed the best stabilization performance of Sb with less pH disturbance.

#### 3.3.2. Column Experiments for Zn and Cu Immobilization

**Figure 6:**
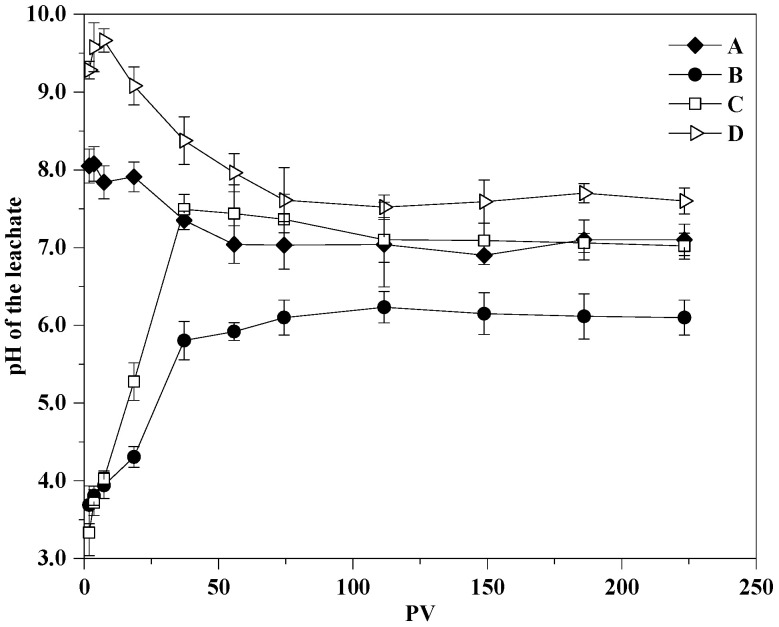
Leach solution pH with change in PV.

The initial Zn (0.056 mg/L) and Cu (0.067 mg/L) concentrations in the leachate of untreated soil in Figure 5c,e, exceeded the GB/T 14848–2017 (Standard for Groundwater Quality) (AQSIQ, 2017) type-I and -II leachate limit, respectively. As shown in Figure 6c–f, the leachate concentration of Zn and Cu decreased significantly when PV < 74.4, showed a slower decrease and tended to stabilize when PV ≥ 74.4. The final leachate concentration of Zn and Cu stabilized at ~0.009 and 0.0035 mg/L, which was 6.2 and 19.1 times lower than that of the initial concentration, respectively. The initial Zn and Cu concentrations in the leachate of soil, amended with 5% FeSO_4_·7H_2_O and 4% Al(OH)_3_, were 7.83 and 0.069 mg/L, which were 139 and 1.04 times higher than those of untreated soil, respectively. After Fe–Al-based adsorbent addition, the soil became transitory acidic, which promoted Zn and Cu leachate [66]. A significant increase in the leachate concentration of Zn and Cu was observed in stabilized soil with acidification, but a significant decrease in the leachate concentration of Zn and Cu was observed in stabilized soil with alkalization, which was the result of the surface charge variation in minerals that were induced by pH and the positively charged property of Zn and Cu [67]. For PV ≥ 74.4, the leachate concentrations of Zn and Cu in the three groups of modified soil were consistent with untreated soil as the pH eventually equilibrated.

The calculation results of the cumulative leachate amounts of Zn and Cu in the dynamic process of each group are shown in Figure 5d, f. The total leachate amount of Zn in Groups A, B, C, and D was 2.88, 44.37, 11.34, and 1.19 mg/kg, respectively. The amount of released Zn increased slowly when PV ≥ 74.4 and depended on the soil pH [73]. The total amounts of Cu leached in Groups A, B, C, and D were 1.27, 2.02, 1.55, and 1.23 mg/kg, respectively. The highest release resulted in Fe–Al-stabilized soil with the acidification group. In the four groups, even a moderate change in pH could induce persistent Cu release.

#### 3.3.3. pH Effects on Sb and Co-Occurring Heavy Metals Immobilization

The initial leachate pH for a 5% FeSO_4_·7H_2_O + 4% Al(OH)_3_ addition with or without a pH regulator differed significantly from the pH of the control groups; however, pH differences between the groups decreased to PVs up to 55.4 (Figure 5). The initial pH values of Groups A and D were 8.05 and 9.30; these values decreased with an increase in PV, and the pH of the column leachate tended towards stability when the PV reached 74.4. In contrast, the initial pH values in the leachate of Groups B and C were 3.70 and 3.30, which increased with the increase in PV to 37.2 and 74.4. The dynamic column experiment results show that the leachate solution pH stabilized at 6–8. The addition of 5% FeSO_4_ and 4% Al(OH)_3_ without a pH regulator had less effect on the final column soil pH.

The change in pH with PV affected Sb, Zn, and Cu stabilization in the soil. Sb could be amended by Fe–Al-based mixed amendments in a wide pH range (pH = 3–10) by strong binding to Fe oxyhydroxides as inner-sphere surface complexes [74] and the optimization pH range for adsorption was less than 7. This result agreed with the findings of Leuz et al. [32], who found that H^+^ could increase the positive surface charge of iron oxyhydroxides and enhance the sorption capacity of Sb(OH)_6_^-^. However, when the pH exceeded 8, Sb(V) desorption was observed in the study of Leuz et al. [32]. This desorption reaction occurred mainly because the surface electronegativity of alkaline soil exceeded that of acidic soil, and it had a strong repulsive force to the anion Sb(OH)_6_^-^, which was not conducive to adsorption [75]. Therefore, the retention and stabilization efficiency of Sb decreased with an increase in pH. In contrast to Sb, the stabilization efficiency of Zn and Cu in soil increased significantly with an increase in pH. The stabilization of Zn and Cu under alkaline conditions was better than that under acidic conditions. This reduced retention effectiveness in acidic conditions could be caused by the following: First, H^+^ replaces Zn^2+^ and Cu^+^ in the complex that is formed by the adsorption reaction, which induces Zn and Cu ions to migrate into the soil pore water [76]. Second, the partial dissolution of the sorbent, especially Al(OH)_3_, in the soil leads to reduced sorption sites and potentially a lower retention of Zn and Cu. However, because of limited Sb sorption capacity, the partial dissolution of Al(OH)_3_ had little effect on Sb adsorption under acidic conditions [4]. This pH contrast between Sb stabilization and co-occurring metals highlights the immobilization advantages of FeSO_4_-mixed Al(OH)_3_ addition.

## 4. Conclusions

This work shows that the studied Fe–Al-based amendments are suitable for the simultaneous stabilization of Sb and co-occurring metals, such as Cu and Zn in primary explosives-sites soil that is contaminated with a high concentration of Sb. Experimental results show that FeSO_4_·7H_2_O had an ideal retention of Sb and Al(OH)_3_ and could prevent Cu and Zn mobilization. Batch and column leachate results showed that a 5% FeSO_4_·7H_2_O + 4% Al(OH)_3_ application mixed with soil could immobilize Sb and retain co-occurring metals in highly contaminated Sb soils. The results of pH-regulated column tests indicated that acid conditioning favored Sb retention. Amendment addition had a positive impact on Sb retention and reduced the labile fractions of Sb. However, the retention behavior and mechanism, ion-exchange mechanism involved in these processes, and possible changes in remediation effect with time must be determined from further experiments. Amendment selection for primary-explosives-contaminated soils is based primarily on its ability to reduce the concentration of labile Sb, Cu, and Zn. In contrast, additional effects, such as the impact of amendments on the soil microbial abundance, community, diversity, and soil functionality are often neglected. Hydrolytic reactions of iron salt can cause soil acidification, and consequently limit site utilization. Sulfate addition to soil from FeSO_4_·7H_2_O likely had a substantial influence on the microbial community. These changes may affect key soil ecosystems and result in an ecological risk in the amended soil. The soil ecological risk must be investigated further after the addition of these efficient chemical amendments.

## Figures and Tables

**Table 1:** Main physicochemical characteristics of soils.

Soil	pH	Concentrations (mg/kg)				
		Mn	Fe	Cu	Zn	Sb_tot_
S1	8.37 ± 1.70	499 ± 66.54	32,200 ± 876.43	25.37 ± 0.17	71.19 ± 14.81	26.73 ± 2.31
S2	7.8 ± 0.27	573 ± 36.67	30,000 ± 156.47	24.29 ± 3.12	67.62 ± 0.52	61.32 ± 4.27
S3	8.11 ± 0.75	545 ± 20.82	28,900 ± 488.27	33.55 ± 1.57	83.80 ± 3.50	108.01 ± 6.43
S4	7.96 ± 0.19	413 ± 41.93	24,200 ± 893.30	232.85 ± 5.87	402.75 ± 95.65	216.60 ± 9.64
S5	7.69 ± 0.40	628 ± 16.05	31,200 ± 366.70	186.74 ± 2.75	868.02 ± 78.23	267.26 ± 172.5
S6	7.93 ± 1.41	396 ± 14.83	23,000 ± 259.53	103.76 ± 5.44	331.36 ± 11.83	512.09 ± 19.52
S7	8.15 ± 1.58	513 ± 37.53	25,000 ± 456.95	312.30 ± 14.83	981.60 ± 10.42	719.36 ± 23.34
S8	7.91 ± 0.08	554 ± 52.51	23,600 ± 99.28	35.26 ± 1.56	215.88 ± 7.25	953.91 ± 39.72
S9	8.25 ± 1.87	719 ± 189.57	20,000 ± 127.71	80.16 ± 3.05	1330.05 ± 36.67	4255.03 ± 231.50
